# Consequences of scarcity: the impact of perceived scarcity on executive functioning and its neural basis

**DOI:** 10.3389/fnins.2023.1158544

**Published:** 2023-06-13

**Authors:** Long Huang, Xiaojuan Li, Fuming Xu, Fuhong Li

**Affiliations:** ^1^School of Humanities and Management, Wannan Medical College, Wuhu, China; ^2^Faculty of Education Yunnan Normal University, Kunming, China; ^3^School of Education Science, Nanning Normal University, Nanning, China; ^4^School of psychology, Jiangxi Normal University, Nanchang, China

**Keywords:** flexibility, perceived scarcity, electroencephalograph, executive functioning, neural basis

## Abstract

**Introduction:**

Previous studies have found a causal relationship between scarcity and the adverse impact it has on executive functioning. However, few studies have directly examined perceived scarcity, and cognitive flexibility (the third component of executive functions) has rarely been included.

**Methods:**

Using a 2 (group: scarcity group vs. control group) × 2 (trial type: repeat trial vs. switch trial) mixed design, this study directly explored perceived scarcity’s impact on cognitive flexibility and revealed its neural basis in the switching tasks. Seventy college students participated in this study through open recruitment in China. A priming task was used to induce perceived scarcity, thus exploring the impact of perceived scarcity on participants’ performance in switching tasks and enabling the analysis of the neural activity of the brain, combined with electroencephalograph (EEG) technology.

**Results:**

In terms of behavioral outcomes, perceived scarcity led to poorer performance and a greater switching cost of reaction time in the switching tasks. Regarding neural activity, perceived scarcity led to an increase in the amplitude of P3 differential wave (repeat trials minus switch trials) in the parietal cortex during the target-locked epochs in the switching tasks.

**Discussion:**

Perceived scarcity can lead to changes in the neural activity of the brain regions related to executive functioning, resulting in a temporary decrease in cognitive flexibility. It may lead to individuals unable to adapt well to the changing environment, unable to quickly devote themselves to new tasks, and reduce work and learning efficiency in daily life.

## 1. Introduction

Compared to past periods of famine and drought (Chakravarthy and Booth, 2004), current material resources are generally very rich. However, many people still live in poorer regions and countries, including developed countries, and still face chronic resource scarcity (Banerjee and Duflo, 2007). Even those living in relative abundance often feel that certain resources are insufficient to meet their needs (Banerjee and Duflo, 2007; Hill et al., 2012; Mullainathan and Shafir, 2013). Recently, the global COVID-19 pandemic has exacerbated the scarcity of related resources such as money, time, and freedom to a certain extent. Generally, a large percentage of people worldwide have experienced some type of scarcity (e.g., time, money, or emotions) at some point in their lives. Scarcity thus remains a pervasive part of our daily lives (Cialdini, 2009; Griskevicius et al., 2013; Fernbach et al., 2015), and issues related to scarcity are often considered, worried about, and discussed (Twist and Barker, 2006).

Resource scarcity often leads to a perceived scarcity of resources. In fact, regardless of whether objective resources are truly scarce, when a person perceives their resources to be less than what they feel they need, perceived scarcity has already arisen (Mullainathan and Shafir, 2013). Even objectively wealthy people often experience perceived scarcity due to their needs not being fully met (Yamauchi and Templer, 1982). Subjective and objective scarcity are thought to affect people’s cognition, choices, and behavior in remarkably similar ways (Mullainathan and Shafir, 2013). Particularly, the impact of monetary scarcity on cognitive functioning has become a research focus in the field of psychology (Mullainathan and Shafir, 2013; Cannon et al., 2019). Researchers believe that scarcity causes individuals to devote excessive attention and cognitive resources to scarcity-related issues, which, in turn, leads to cognitive load and a decline in executive functioning (Mullainathan and Shafir, 2013). This result seriously hinders people’s daily lives, causing them to behave irrationally, such as through attention neglect, impulsivity, memory loss, and irrational decision-making, among others (Zhao and Tomm, 2018; Ong et al., 2019).

### 1.1. Scarcity theory

Mullainathan and Shafir (2013) define scarcity as “having less than you feel you need” (p. 4). They used scarcity theory to explain the cognitive and behavioral changes in people’s lives when facing scarcity of a certain resource. This theory builds on cognitive psychology, which studies the characteristics of the cognitive processes that influence human behavior. The core idea of scarcity theory is that scarcity induces a specific state of mind by affecting people’s thinking, which in turn affects their cognition and behavior.

Scarcity theory argues that scarcity affects decision making and behavior through two core psychological mechanisms: tunneling and cognitive load. First, scarcity heightens focus on addressing scarcity-related issues, thereby increasing resource efficiency, and promoting memory encoding. However, ignoring issues unrelated to the scarcity leads to forgetfulness, neglect, and excessive borrowing (Zhao and Tomm, 2018; Ong et al., 2019). This process of focusing and ignoring is called “tunneling.” Second, attention and scarcity issue-related thoughts create a bandwidth load (a metaphor for cognitive load) and reduce mental bandwidth. This leads to a decrease in the individual’s cognitive and executive control abilities, which makes it impossible to resist the temptation of instant gratification, and thus increases the time discount rate. The scarcity theory argues that cognitive load is the basis for scarcity’s negative impact on cognitive ability and executive functions.

Many existing studies support scarcity theory to some extent. A series of laboratory studies was published in science by Shah et al. (2018) that manipulated opportunity scarcity, time scarcity, and economic scarcity through games such as “WoF.” These studies found that although resource-deprived participants were in the game for less time due to resource scarcity, they felt more fatigued and took more time to make decisions during the task. Simultaneously, participants performed worse on subsequent attention tests with more non-interest-rate borrowing, which in turn led to worse game performance. In the final experiment, participants were able to preview the next round of questions at the bottom of the screen while viewing the current question. Results indicated that participants experiencing scarcity focused more attention on the problem in their current round. Despite being able to preview the next round’s questions, they had no time to preview or think about them. Participants who were not experiencing scarcity could consider the profit and loss of the next round in advance, while also considering the current round. Therefore, the scarcity of any one resource can lead to excessive attention being devoted to related tasks, which can lead to the neglect of other stimuli.

In Spears (2011) study, participants were divided into a scarcity group and a rich group by allowing them to choose either one or two commodities from three commodities. The scarcity group could choose one of the three items, while the rich group could choose two of the three. Half of the participants in each group could actively choose, while the other half only passively accepted their choices. The participants then performed grip and Stroop tasks to measure their inhibitory control ability. The results indicated that participants in the active choice scarcity group had significantly lower inhibitory control than those in the passive acceptance scarcity group because the participants in the active choice group used more cognitive resources during the initial commodity selection task. Carvalho et al. (2016a,b) further differentiated the impact of long-term and short-term economic conditions on inhibitory control ability and found that long-term financial stability was associated with a willingness to wait for higher returns and an increase in inhibitory control ability. Participants also showed it harder to resist instant gratification on paydays than afterwards, given the temporary change in economic conditions around these days. Baumeister et al. (1994) argue that shifting attention from oneself to the environment leads to a depletion of self-control. Mann and Ward (2007) found that when individuals want to divert their limited attentional resources from their immediate needs to distant stimuli, this also leads to greater depletion of executive functioning.

In summary, we can infer that scarcity can affect cognitive functioning through attention, cognitive load, and other mechanisms, and can temporarily reduce individuals’ performance on cognitive tasks such as intelligence tests, inhibitory control tasks, and memory tasks. However, previous empirical research still focused on the scarcity of objective resources, ignoring research on subjective perceived scarcity. Previous research suggests that both objective scarcity and subjective perceived scarcity affect human cognition, choice, and behavior in similar ways (Mullainathan and Shafir, 2013). However, the measurement of subjective scarcity is still necessary. Because the nature of subjective perceived scarcity and objective scarcity differ, objective resource scarcity is absolute and relative, whereas perceived scarcity is relative and subjective. Individuals with objective and subjective scarcity do not completely coincide. Additionally, recent laboratory studies have explored the impact of incidental scarcity on executive functioning in the general population by creating scarcity scenarios but without precise measurement of participants’ subjective perceived scarcity. Therefore, it is necessary to explore the impact of perceived scarcity on cognitive functioning.

The manipulation of perceived scarcity in the laboratory is the key to exploring the causal relationship between perceived scarcity and other variables. Researchers have designed and validated some mature and reliable manipulation paradigms. The first type of manipulation is the recall task, which effectively activates an individual’s perceived scarcity by asking participants to recall past scarce experiences (Roux et al., 2015). Some researchers also asked participants to recall the items they most wanted but were unable to purchase due to a lack of money, and to think about how to solve this problem and the adverse effects it may have on their lives. This manipulation is believed to effectively activate an individual’s perceived scarcity (Benjamin et al., 2013). The second type of manipulation is concept initiation. The concept of resource scarcity can also be initiated through some implicit techniques. Previous studies have shown that words related to scarcity make it easier for individuals to associate scarce related content. The researchers asked participants to watch words, images (such as photos of impoverished families) (Durante et al., 2015), and news (Durante et al., 2015) related to scarcity (such as scarcity, resources, sparsity, limited) presented on the screen (Krosch and Amodio, 2014), thereby inducing individuals’ perceived scarcity of resource. Researchers also use heterotopic word formation to disrupt the order of words related to scarcity, allowing participants to recognize and spell correct words, thereby inducing individuals’ perceived scarcity of resource (Rodeheffer et al., 2013). In addition, some previous studies often used situational and game tasks to manipulate individuals’ perceived scarcity (Sharma et al., 2014; Sze et al., 2017). In the current research, we combine recall task and concept initiation methods to activate individuals’ perceived scarcity.

### 1.2. Executive functioning and cognitive flexibility

Executive functioning, also known as executive control or cognitive control, refers to the ability to retain information in working memory, inhibit an individual’s unthinking reactions to stimuli, and flexibly switch an individual’s cognitive focus. It is the basis for an individual’s conscious self-direction and control of their own behavior (Blair, 2016). Executive functioning helps us limit impulsive reactions, regulate emotions, and avoid making bad decisions with short-term benefits and long-term disadvantages. Executive functioning can help us perform well in school, life, and work, solving specific problems and planning ahead in real life, making our lives smoother (Blair, 2016). Executive functions include three core competencies: inhibitory control, working memory, and cognitive flexibility (Miyake et al., 2000; Lehto et al., 2003).

Previous empirical studies of scarcity’s impact on executive functioning have mainly focused on two aspects: inhibitory control and memory. Only a few researchers have investigated the impact of scarcity on cognitive flexibility (third executive function). Cognitive flexibility builds on two other abilities (inhibitory control and working memory) (Davidson et al., 2006; Garon et al., 2008; Diamond, 2013) and is more reflective of integrated executive functioning. The task-switching paradigm is often used in the laboratory to study individuals’ cognitive flexibility. The classic task switching paradigm often involves two tasks. Participants are required to respond to the task according to task rules and clues. All trials are divided into switch or repeat trials according to the similarities and differences between the two consecutive trials (Diamond, 2013). Common behavioral outcome measures in the task-switching paradigm include reaction time and error rate for repeat trials, reaction time and error rate for switch trials, and the switching cost of reaction time and error rate. The switching cost is the difference between the reaction time (and error rate) of the switching trials and the reaction time (and error rate) of the repeat trials. Switching costs are used as an operational definition of cognitive flexibility (Meiran et al., 2000; Miyake et al., 2004; Friedman et al., 2008). Smaller switching costs indicate greater cognitive flexibility and executive functioning. Existing empirical studies have found that scarcity can lead to a decrease in executive functions such as inhibitory control and memory (Mani et al., 2013; Zhao, 2014). When inhibitory control and working memory decline, cognitive flexibility also declines (Friedman et al., 2008). Recently, Zhang (2020) study on relative deprivation found a resulting decline in individual cognitive flexibility. Based on the similarity between relative deprivation and scarcity, this result supports the relationship between perceived scarcity and cognitive flexibility.

With the development of cognitive neuroscience, electroencephalographic (EEG) research has recently revealed the neural mechanisms of cognitive flexibility to a certain extent (Han et al., 2018). In cue-goal switching tasks, participants’ processing of cues and goals corresponds to different neural reactions (Tieges et al., 2007; Karayanidis et al., 2010; Kieffaber and Hetrick, 2010). Researchers have mainly focused on the P3 components in clue-locked epochs and target-locked epochs. Previous studies have found that the P3 component of clue-locked epochs is thought to be associated with cognitive readiness (Karayanidis et al., 2010), updating the current set of relevant tasks (Kray et al., 2005; Tieges et al., 2007; Kieffaber and Hetrick, 2010), attention shifting (Kieffaber and Hetrick, 2010), and anticipatory activity in executive functioning (Karayanidis et al., 2003; Tieges et al., 2007). Han et al. (2018) found that switch trials showed larger P3 amplitudes in clue-locked epochs than repeat trials. In contrast to the clue-locked epochs, the P3 amplitudes of the target-locked epochs were reversed. Switch trials elicited smaller P3 amplitudes in the parietal cortex of the target-locked epochs than in repeat trials (Karayanidis et al., 2003; Tieges et al., 2007; Kieffaber and Hetrick, 2010). The decrease in P3 amplitude during the target-locked epochs is thought to reflect weak or unstable linkages between the stimulus and task setting in switch trials (Waszak et al., 2003). Therefore, the amplitude of the P3 difference wave (repeat trials minus switch trials) also reflects switching costs. The greater the switching cost, the weaker the connection between the stimulus and task in the switch trial and the larger the P3 difference wave. According to the behavioral results of existing research, it is speculated that perceived scarcity will result in a decline in cognitive flexibility. Thus, the decline in cognitive flexibility caused by perceived scarcity results in an increase in switching cost, which also leads to a larger amplitude of the P3 difference wave in the parietal cortex between the repeat and switch trials.

### 1.3. Summary and research questions

In summary, previous studies have confirmed the impact of scarcity on executive functions such as inhibitory control and memory. However, the focus of existing empirical research is still the objective scarcity of resources, ignoring subjective perceived scarcity. Moreover, empirical studies focus on scarcity’s impact on inhibitory control and memory, and there are few studies that focus on its impact on cognitive flexibility. Additionally, no study has investigated the neural mechanisms by which perceived scarcity affects executive functioning. Accordingly, the current study intends to induce participants’ perceived scarcity by recall tasks and concept initiation tasks and then exploring the impact of perceived scarcity on cognitive flexibility. We combined ERP technology to further explore the neural mechanisms by which perceived scarcity affects cognitive flexibility. Based on existing research, we speculated that perceived scarcity depletes cognitive resources, resulting in decreased cognitive flexibility and increased switching costs (Hypothesis 1). Perceived scarcity also leads to increased amplitude of the P3 differential wave (repeat trials minus switch trials) in the parietal cortex (Hypothesis 2).

## 2. Methods

### 2.1. Data

The current study recruited 70 college students via open recruitment in China. Two of the participants’ data were incomplete, and we excluded these from the sample data during data analysis. The current study thus obtained 68 valid samples (18 males and 50 females, *M* = 19.62, *SD* = 1.51). There were 34 people in the scarcity group and 34 in the control group, of which one person in the scarcity group’s EEG data collection was unsuccessful. All participants reported that they had not participated in similar experiments or had acquired relevant knowledge.

### 2.2. Design

This study used a 2 (group: scarcity group vs. control group) × 2 (trial type: repeat trial vs. switch trial) mixed design. The first independent variable was a between-subjects variable, and a priming task (recall tasks and concept initiation tasks) was used to divide the participants into two levels: the scarcity group and the control group. The second independent variable was the within-subject variable, which consisted of two types of trials in the switching task, including two levels of the repeat trial and switch trial. The dependent variables were the reaction time and error rate in the repeat and switch trials, switching cost of the reaction time and error rate, and amplitudes of P3 difference wave (repeat trials minus switch trials) in the target-locked epochs.

### 2.3. Procedure

Participants were told that they would take a test of cognitive responsiveness, and the entire experiment was conducted using the E-prime software. The main steps include the following: First, participants were randomly assigned to the scarcity or control group and performed a word memory task (concept initiation task) of scarce or neutral words to initiate their concept of scarcity. Second, they performed a switching task during the practice phase. Third, starting with Episodic Recall Task 1, participants in the scarcity group were asked to recall their most money-poor situations and feelings and describe them in maximum detail. Participants in the control group were asked to describe in maximum detail how they spent their evening. Subsequently, participants’ perceived scarcity, mood, and cognitive depletion were measured. Fourth, the participants performed the first block’s formal switching task. Fifth, to maintain the participants’ perceived scarcity, they were asked to perform Episodic Recall Task 2. The scarcity group was asked to recall 2–3 things that they wanted to do but could not because of a lack of money. The control group was asked to describe the names and functions of the buildings they passed from the dormitory to the school gate in maximum detail. Sixth, the participants completed another block’s switching task. Seventh, participants completed a vocabulary recognition task. Finally, the participants completed a general demographic questionnaire and a trait self-control scale.

### 2.4. Measures

#### 2.4.1. Priming tasks

In the current study, considering that the conversion task lasted for 30 min. In order to maintain an individual’s persistent perceived scarcity, the priming tasks that induced perceived scarcity in the study included episodic recall and concept initiation task (vocabulary memory tasks).

In an episodic recall task (Fischhoff et al., 2003; Roux et al., 2015), participants in the scarcity group were first asked to recall a moment when they lacked money and describe in detail the experience and psychological activities at that time. Participants also needed to list some things that they desired but could not achieve due to the lack of money, whether they would take out loans or other methods to achieve those desires, and how this affected their lives. Participants in the control group were asked to recall what they typically did at night and describe their experience in detail. Then, they were asked to list the buildings they would pass by on their way from the dormitory to the school gate, writing down their functions, features, shapes, and so on.

**Figure 1:**
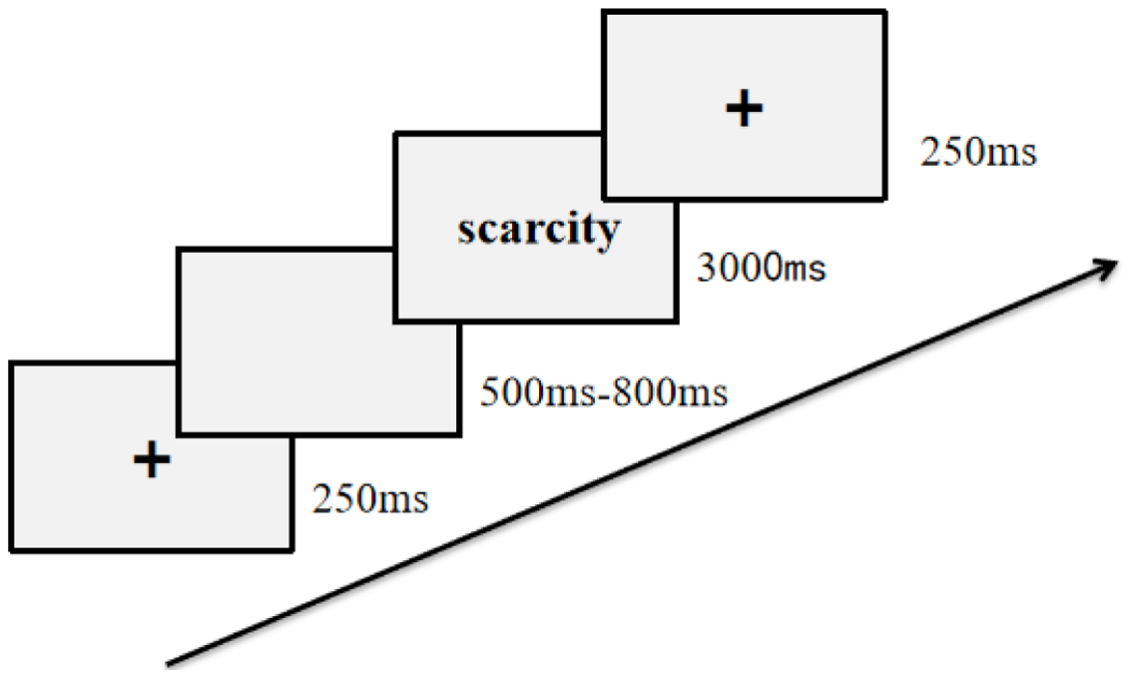
Flow chart of vocabulary memory task.

The vocabulary memory task was adapted from Krosch and Amodio (2014) research, and is believed to be impactive in initiating implicit cognition. Before the switching tasks, participants were presented with 30 words (3 s each) in random order (Figure 1). The participants were asked to memorize as many of these words as possible, thus triggering their perceived scarcity. These words were also represented in the cue part of the switching task to maintain the priming state of the perceived scarcity. To enhance participants’ deep processing of these words. Participants were told that after completing the switching task, they needed to recognize these words again to determine whether they had appeared in the previous memory task. Participants had 3 s to respond to keystrokes for 16 sequential words. Among them, eight words appeared in the memory task and required the “F” key to be pressed, and eight were interference words that had not appeared in the memory task. For the latter, participants were required to press the “J” key. Failure to respond within 3 s, or making a wrong keystroke, was recorded as a failure of recognition.

To verify the implicit priming impact of memorized words on perceived scarcity, we conducted a preliminary experiment. In the pre-experiment, we recruited 87 additional college students (37 males and 50 females, *M* = 20.94, *SD* = 1.91) who were asked to describe how well the presented words reminded them of the concept of scarcity. A 7-point scoring system was used, with “1” representing “completely disagree” and “7” representing “completely agree.” The paired sample *t*-test results showed that the score of the 30 scarce words (*M* = 4.83, *SD* = 1.30) was significantly higher than that of the 30 neutral words (*M* = 3.54, *SD* = 1.81) when associated with scarce concepts, *t* = 6.60, *p* < 0.001, Cohen’s *d* = 0.82. The scarcity words can thus activate the individual’s perceived scarcity.

#### 2.4.2. Switching task

**Figure 2:**
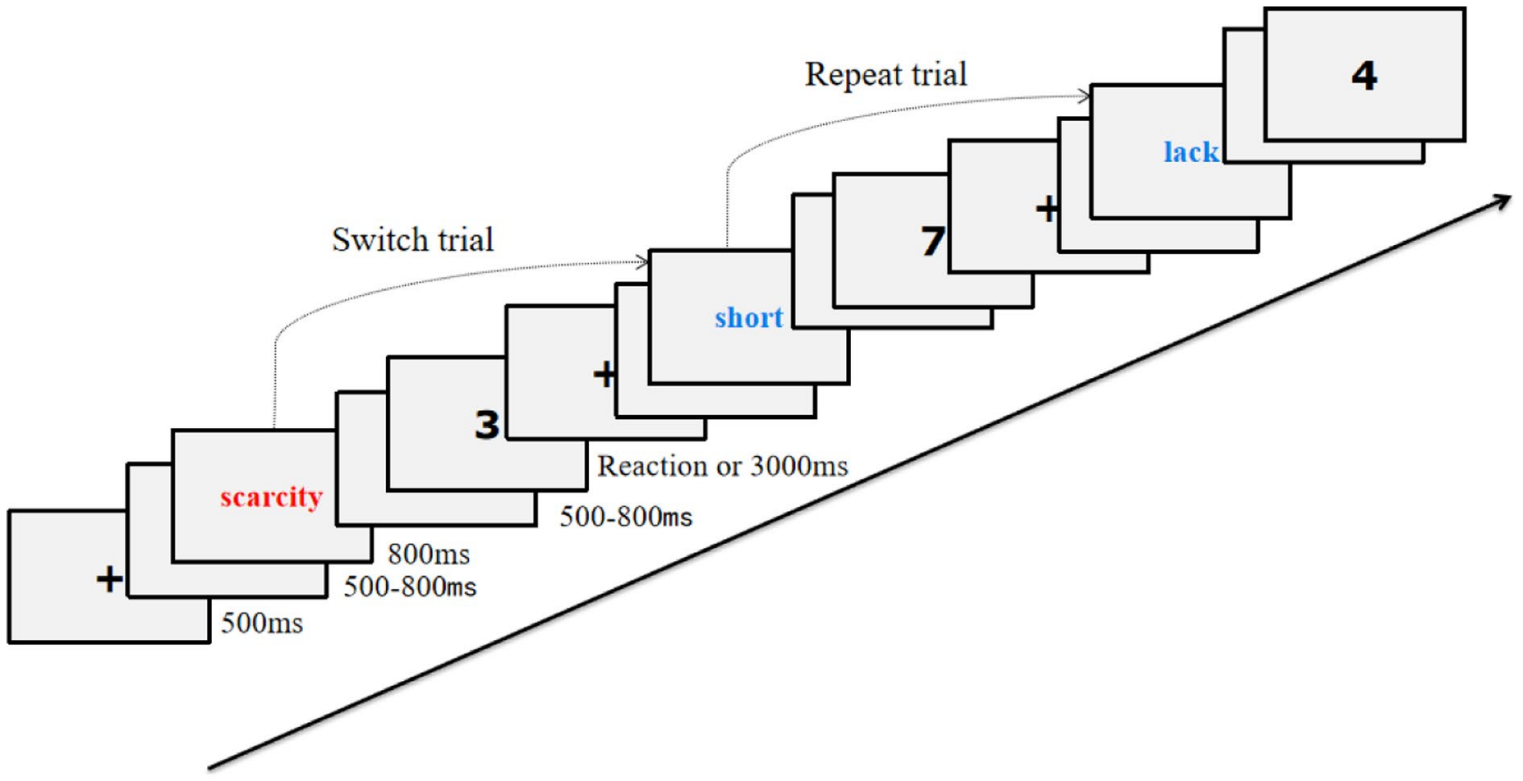
Flow chart of switching task paradigms.

In order to explore the cognitive flexibility of participants, we adapted the task-switching paradigm. In this paradigm, we presented cues and target stimuli separately and successively (Figure 2). A 500 ms fixation point (“+”) was presented first, followed by a random temporal blank screen of 500–800 ms, then an 800 ms cue stimulus appeared consisting of conceptual priming words. This cue stimulus indicated to the participant which task to perform, after which the participant was presented with another randomly timed blank screen of 500–800 ms. This was followed by a target stimulus (1, 2, 3, 4, 6, 7, 8, or 9). Participants responded to the target stimuli according to the task rules corresponding to the cue stimuli and were presented with the content of the next trial after they had responded or for 3,000 ms.

There are two reaction tasks in this paradigm, including size (compared to 5) or parity judgments for the eight numbers “1, 2, 3, 4, 6, 7, 8, and 9.” When the presented cue stimulus was a red word, participants were required to perform a size-judgment task for the subsequent target stimulus. If the number was greater than five, they had to press the “F” key, if the number was less than five, they had to press the “J” key (the keys were balanced among subjects). When the cue stimulus was a blue word, it was necessary to perform the parity judgment task on numbers. Participants pressed the “F” key for odd numbers and the “J” key for even numbers. The color of the clues in the repeat trial was the same as the colors in the previous trial, and the same judgment task was performed on both trials. For example, if both the n-1 and n trial cued stimuli were red, participants were asked to judge the “size” of the number in two consecutive trials.

Conversely, the switch trial presented cues that had different colors from the n-1 trial so that participants would perform different tasks in the n and n-1 trials. For example, if the cued stimulus was red in the n-1 trial and the cued stimulus was blue on the n trial, then participants needed to make a size judgment on the n-1 trial, and make an “odd-even” judgment on the n trial. The entire study included a formal experiment of two blocks and a practice experiment of one block. The practice block included 10 trials. When the specified rate of the participants reached 90% in the 10 practice trials, the participants started the formal experiment; otherwise, they continued to practice. The formal experiment contained two blocks, each of which consisted of 122 trials, of which the first two trials did not undergo statistical analysis. Of the 120 subsequent trials, 60 were repeat trials, and 60 were switch trials. The order of all trials was pseudo-random, and participants did not know in advance whether the trials they faced would be repeat or switch trials.

#### 2.4.3. Scarcity scale

The resource scarcity perception questionnaire, adapted from Roux et al. (2015), was used to examine whether the manipulation of the priming task to induce participants’ perception of financial scarcity was successful. The entry was as follows: I do not feel as if I have enough money right now. A 7-point Likert scale was used to subjectively rate their compliance with scarcity status (1 = strongly disagree, 7 = strongly agree). A higher score indicated higher perceived scarcity.

#### 2.4.4. Self-assessment cognitive depletion scale

The current study refers to the three self-assessment items used in existing studies to measure participants’ cognitive depletion after completing the switching task (Dou et al., 2014). The specific items were: (1) How tired are you in this task? (2) How much effort did you put into this task? (3) After completing this task, to what extent do you feel that your energy resources have been depleted? A 7-point scoring system was used to measure the participants’ self-assessment of cognitive depletion, with 1 representing “nothing at all” and 7 representing “very much.” The higher the average score, the greater the cognitive resource depletion.

#### 2.4.5. Trait self-control scale

The current study used the Chinese version of the Self-Control Scale for College Students compiled by Tangney et al. (2004) and revised by Tan and Guo (2008) to measure the subjects’ trait self-control. The scale consisted of 19 items and five dimensions. Specifically, six items measured an individual’s impulse control ability, three items measured the ability to focus on work, four measured the ability to resist temptation, three measured healthy habits, and three measured the ability to moderate entertainment. A 5-point scoring system was used, with 1 indicating “complete non-conformity” and 5 indicating “complete agreement.” Higher scores indicate higher levels of the trait self-control.

### 2.5. Experimental equipment and data processing

Electrophysiological signals were measured using EEG with a sampling frequency of 1,024 Hz and impedance of 5 kΩ or less during recording. The EEG recorded the arrangement of 64 electrode points, which were placed according to the International 10–20 expansion system using elastic caps (Brain Product, GmbH, Germany). The online reference electrode was FCz and the ground electrode was AFz. Two electrodes were placed on the lateral side of the left and right eyes (HEOG) for horizontal EOG recording and one electrode was placed under the right eye to record the vertical EOG. The voltage was amplified by a low-noise electrode differential amplifier with a frequency reaction of 0.01–100 Hz. Digitized signals were recorded using brain visual recording software (Brain Products GmbH, Munich, Germany). Data analysis was performed using the Brain Vision Analyzer 2.1 software. The digitized signal was filtered using a 24-bit analog-to-digital converter with a bandpass of 0.10–30 Hz (24 dB/oct). Eye movements were removed or corrected using an independent component analysis method implemented using brain vision analysis software (Brain Products, GmbH, Germany). Muscle artifacts and other artifacts were removed with a horizontal threshold of ±100 and ± 50 μV as rejection criteria, and 24 Hz low-pass filtering was performed. Baseline corrections were made between 200 and 0 ms before the onset of the target stimulus. Timeframe-locked ERPs were extracted from consecutive EEG recordings, from 200 ms before to 1,000 ms after the stimulus. EEGs from trials of the same type were then averaged according to grouping (scarcity vs. control) and trial type (repetition and switching) to create the ERPs for each electrode.

**Figure 3:**
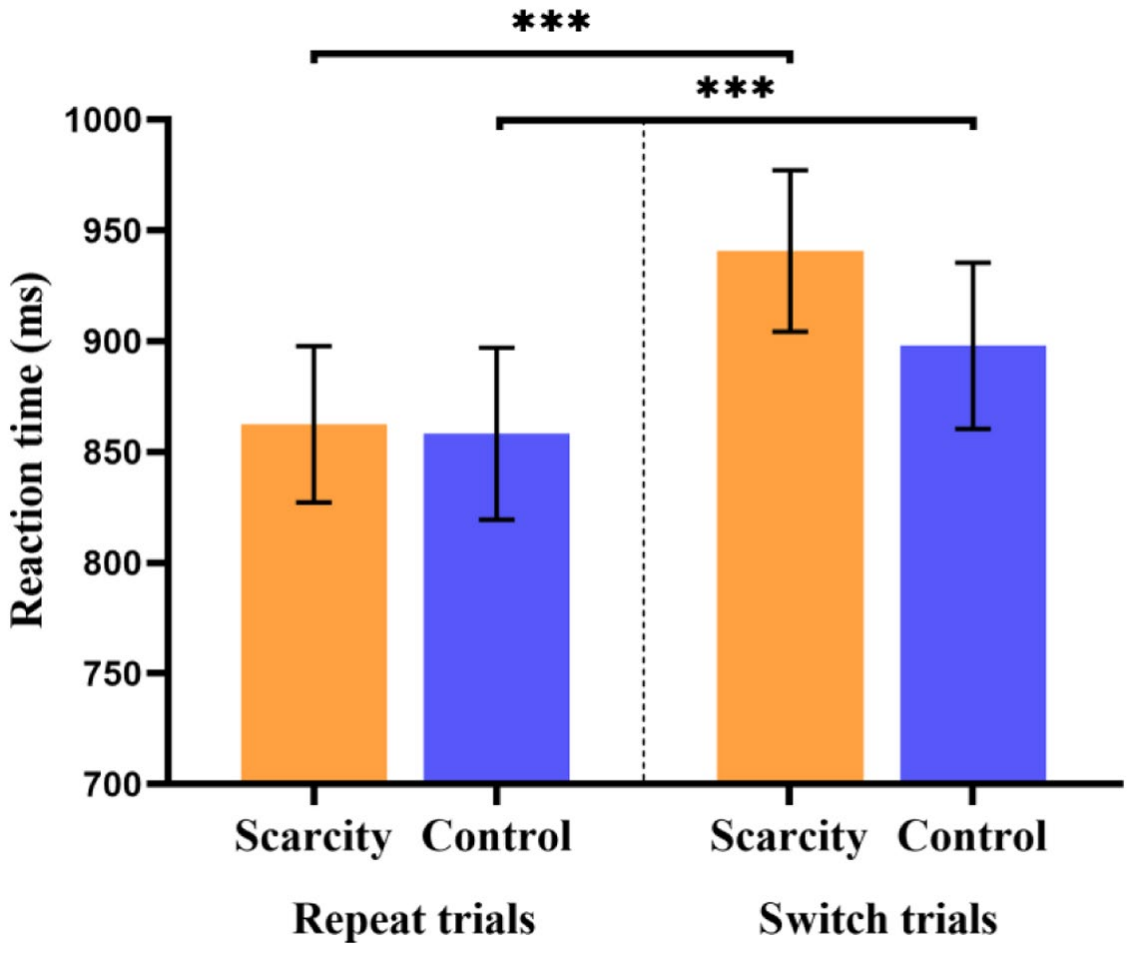
Reaction time of scarcity group and control group in different trial types (***p < 0.001).

As the current study was designed with the addition of scarce or neutral words to cued stimuli, this interfered with the individual’s electrical activity when processing repetitive or switching cues. Therefore, the current study’s focus was the ERP component of undisturbed target locking. The ERP waveforms of the target-locked epochs in different brain regions was drawn as shown in Figure 3, which involved 20 electrode points, including the left frontal cortex (F1/F3/F5/FC1/FC3/FC5), right frontal cortex (F2/F4/F6/FC2/FC4/FC6), left parietal cortex (P1/P3/P5/P7), and right parietal cortex (P2/P4/P6/P8). Based on existing literature (Barceló et al., 2003; Han et al., 2018), combined with the total waveform, brain topography, and peak detection results in the current study, the selected measurement windows were P3 (350–420 ms) in target-locked epochs. The statistical indicator of P3 window was the average peak value of the ERP waveform. A two-factor repeated measures ANOVA of 2 (grouping: scarcity group, control group) × 2 (hemisphere: left parietal, right parietal) was performed on the P3 difference wave (repeat trials minus switch trials), which could reflect the switching cost. SPSS 23.0 was used to process and analyze the data in this study, and the Greenhouse–Geisser method was used to correct the data that did not meet the spherical assumption in the analysis.

## 3. Results

### 3.1. Perceived scarcity group manipulation test

The grouping manipulation of perceived scarcity in this study was tested. An independent samples *t*-test found that the perceived scarcity of the scarcity group (*M* = 5.65, *SD* = 1.45) was significantly higher than that of the control group (*M* = 2.27, *SD* = 1.33), *t*_(67)_ = 10.00, *p* < 0.001, Cohen’s *d* = 2.43. The grouping manipulation of perceived scarcity was thus successful.

### 3.2. Control variables

In order to exclude the influence of the individual’s objective economic status and trait self-control on cognitive flexibility. We analyzed differences in participant’s monthly living expenses and trait self-control level across groups. An independent sample *t*-test was performed on the monthly living expenses of the study’s participants. No significant difference was found between the monthly living expenses (RMB) of the scarcity group (*M* = 1460.29, *SD* = 412.27) and the control group (*M* = 1525.00, *SD* = 297.53), *t*_(67)_ = 0.74, *p* = 0.461. Therefore, the influence of objective economic status on cognitive flexibility was excluded. An independent sample *t*-test was performed on the self-assessed trait self-control level of the study’s participants. The results indicated that there was no significant difference between the self-assessment score for the trait self-control of the scarcity group (*M* = 3.28, *SD* = 0.53) and that of the control group (*M* = 3.21, *SD* = 0.56), *t*_(67)_ = 0.53, *p* = 0.602. Therefore, the influence of trait self-control on cognitive flexibility was excluded.

### 3.3. Self-assessment cognitive depletion

An independent samples *t*-test was performed on the self-assessed cognitive depletion of the scarcity and control groups. The results showed that the degree of self-assessed cognitive depletion in the scarcity group (*M* = 4.09, *SD* = 1.10) was significantly higher than in the control group (*M* = 3.21, *SD* = 1.12), *t*_(67)_ = 3.29, *p* < 0.01, Cohen’s *d* = 0.79. Participants in the scarcity group thus felt that they were depleting more cognitive resources than those in the control group.

### 3.4. Behavioral results

#### 3.4.1. Reaction time

Repeated-measures ANOVA was performed on the reaction times of switch and repeat trials in switching tasks, with grouping (scarcity group or control group) as the between-subject variable, trial type (switching or repeated) as the within-subject variable, as well as reaction time (ms) as the dependent variable (see Figure 3). The results showed that the main impact of the group was not significant [*F*_(1, 67)_ = 0.21, *p* = 0.652]. The main impact of trial type was significant [*F*_(1, 67)_ = 61.38, *p* < 0.001, *η*^2^ *_p_* = 0.482], and the reaction time of the switch trial (*M =* 919.36, *SD* = 215.38) was significantly greater than that of the repeat trial (*M* = 860.33, *SD* = 214.62). The interaction impact of the group and trial type was also significant [*F*_(1, 67)_ = 6.55, *p* < 0.05, *η*^2^ *_p_* = 0.090]. Further simple impact analysis found that participants’ average reaction time in the switch trial (*M* = 940.76, *SD* = 212.89) was significantly greater than participants’ average reaction time in the repeat trial (*M* = 862.45, *SD* = 205.63) in the scarcity group, *p* < 0.001. The participants’ average reaction time in the switch trial (*M* = 897.95, *SD* = 218.89) was also significantly greater than that of participants in the repeat trial (*M* = 858.21, *SD* = 226.33) in the control group (*p* < 0.001). Both the scarcity and control groups thus had switching costs of reaction time in the switching tasks. Additionally, there was no significant difference in reaction times between the scarcity group (*M* = 862.45, *SD* = 205.63) and the control group (*M* = 858.21, *SD* = 226.33) in the repeat trial (*p* = 0.936). In the switch trial, the reaction time of the scarcity group (*M* = 940.76, *SD* = 212.89) was greater than that of the control group (*M* = 897.95, *SD* = 218.89), but the difference was not significant (*p* = 0.417).

#### 3.4.2. Error rate

**Figure 4:**
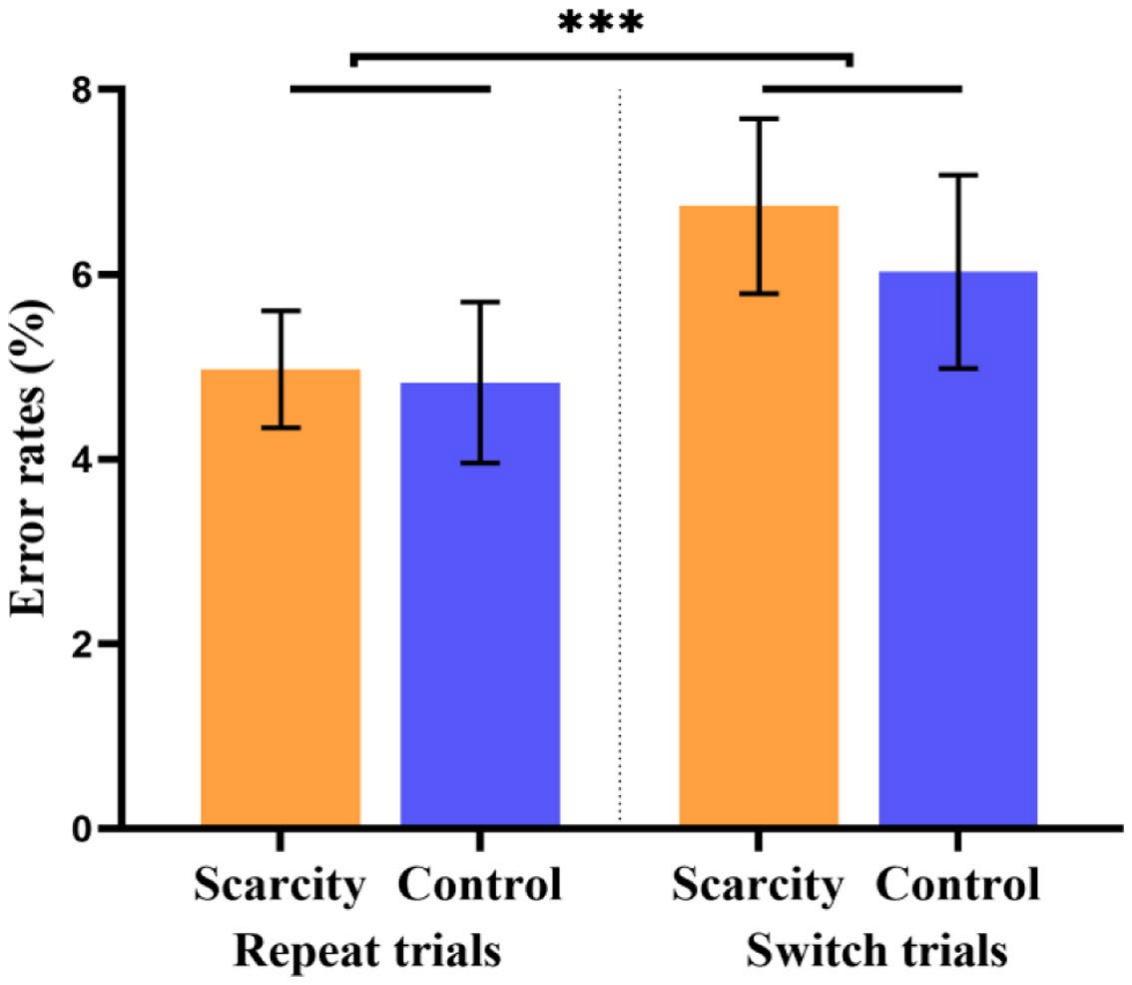
Error rates of scarcity group and control group in different trial types (***p < 0.001).

Repeated-measures ANOVA was performed on the error rates (%) of switch trials and repeat trials in the scarcity and control groups, with grouping as the between-subjects variable, trial type (switch or repeat) as the within-subjects variable, and error rate (%) as the dependent variable (see Figure 4). The main impact of the group was not significant [*F*_(1, 67)_ = 0.13, *p* = 0.720]. The main impact of the trial type was significant [*F*_(1, 67)_ = 13.91, *p* < 0.001, *η*^2^ *_p_* = 0.174]. The error rate of the switch trials (*M* = 6.38, *SD* = 5.79) was significantly higher than that of the repeat trials (*M* = 4.90, *SD* = 4.41) for all participants. The switching cost of the error rate thus exists generally. The interaction effect of the group and trial type was not significant [*F*_(1, 67)_ = 0.50, *p* = 0.481].

#### 3.4.3. Switching cost

**Figure 5:**
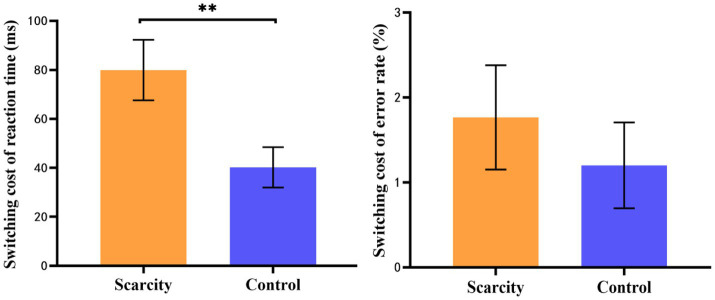
Switching cost of reaction time and error rate in scarcity and control groups (**p < 0.01).

The independent sample *t*-test was carried out with the switching cost (ms) of reaction time and the switching cost (%) of error rate as the dependent variables as well as grouping as the independent variable, as shown in Figure 5. The results showed that the switching cost of reaction time in the scarcity group (*M* = 79.94, *SD* = 72.04) was significantly greater than that in the control group (*M* = 40.22, *SD* = 48.01), *t*_(67)_ = 2.68, *p* < 0.01, Cohen’s *d* = 0.65. While the participants’ switching cost of error rate in the scarcity group was greater than that of the control group, there was no significant difference between the two groups [*t*_(67)_ = 0.71, *p* = 0.481].

### 3.5. EEG results

**Figure 6:**
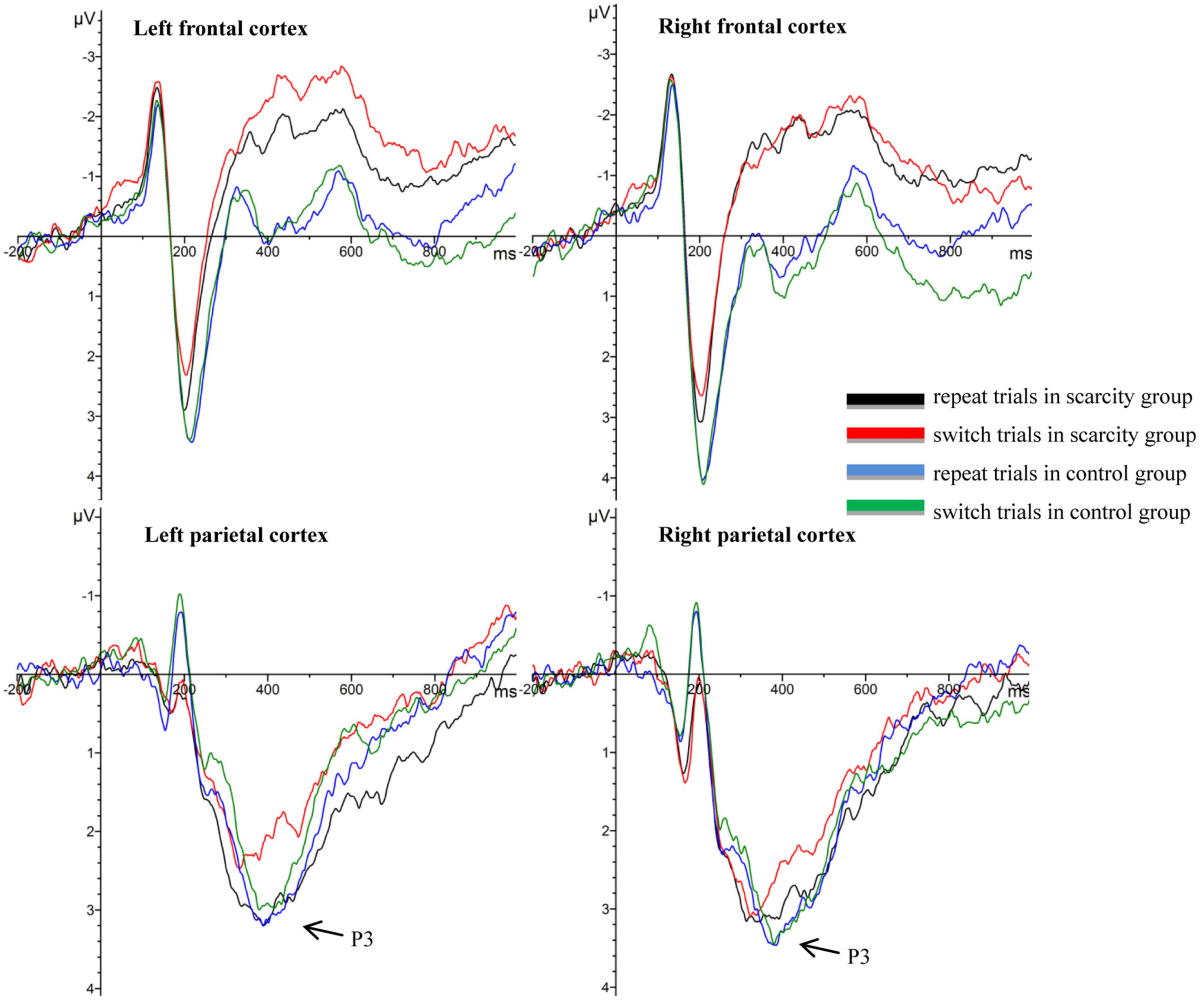
Waveforms of the target-locked epochs.

**Figure 7:**
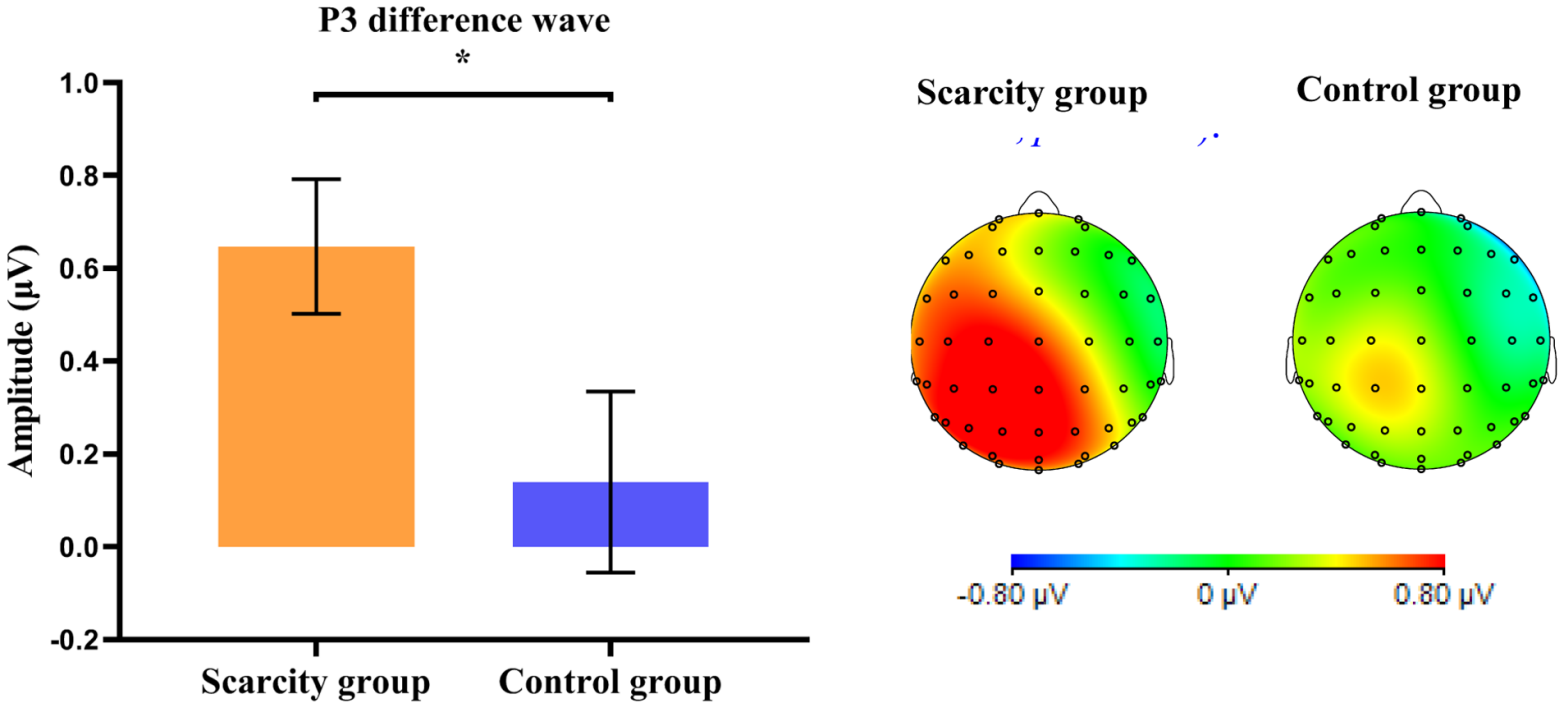
Amplitude of the P3 differential wave (repeat trial minus switch trial) evoked in the parietal cortex and wave topography (350–420 ms) (*p < 0.05).

A two-factor repeated-measures ANOVA was performed on the amplitude (μV) of the P3 difference wave (repeat trials minus switch trials) in the parietal cortex, with grouping as the between-subject variable and hemisphere as the within-subject variable (see Figures 6, 7). The results showed that the main impact of the group was significant [*F*_(1, 67)_ = 4.34, *p* < 0.05, *η*^2^ *_p_* = 0.063]. Participants in the scarcity group (*M* = 0.65, *SD* = 0.83) had significantly larger amplitudes of P3 differential waves in the parietal cortex than those in the control group (*M* = 0.14, *SD* = 1.14). The main impact of the hemisphere was also significant [*F*_(1, 67)_ = 6.77, *p* < 0.05, *η*^2^ *_p_* = 0.094]. The amplitude of the P3 differential waves in the left parietal cortex (*M* = 0.54, *SD* = 1.14) was significantly larger than in the right parietal cortex (*M* = 0.24, *SD* = 1.11). The interaction between the grouping and hemisphere was not significant [*F*_(1, 67)_ = 1.80, *p* = 0.185].

## 4. Discussion

This study found that the switching costs of reaction time and error rate existed in both the scarcity and control groups, which is consistent with previous studies on cognitive flexibility (Kiesel et al., 2010; Koch et al., 2018). Participants’ reaction times were longer in switch trials than in repeat trials, and the error rate in switch trials was higher. This study also found that the participants in the scarcity group reported that they depleted more cognitive resources in the recall task than those in the control group, which confirmed the effect of scarcity on cognitive depletion. More importantly, the reaction-time switching cost of the scarcity group was significantly greater than that of the control group, suggesting that cognitive depletion caused by perceived scarcity can further lead to individuals to exhibit reduced cognitive flexibility in switching tasks. Compared to the control group, participants in the scarcity group’s reaction times were longer in switch trials than in repeat trials. Although there was no significant difference between the scarcity and control groups in the error rate switching cost, there was a similar trend. This is because switch trials are more difficult and require participants to suppress the previous task rules and invoke the new task rules instead. The perceived scarcity of money induces individuals to pay excessive attention to and worry about money-related issues, which leads to the burden of bandwidth, consumes individuals’ limited cognitive resources, and reduces their cognitive flexibility. This makes it more difficult for individuals to suppress previous task rules in switch trials, thus making it more difficult for individuals to quickly use new task rules. Previous studies have suggested that such worries about financial situations have a negative impact on executive functioning, comparable to the consequences of a sleepless night (Linde and Bergström, 1992). The results of the current study are also consistent with some recent studies on simulating financial distress and time scarcity, although they focus on the other two cognitive abilities (inhibitory control and memory). Research published in Science by Mani et al. (2013) also found that participants’ inhibitory control abilities were inversely related to financial distress severity, with a transient decrease in inhibitory control abilities when they resolved financial distress. As economic scarcity impairs cognitive functioning, time scarcity also impairs individual cognitive and behavioral performance. Studies by Spears (2011) and Zhang (2019) also found that manipulating scarce situations in a laboratory can lead to worse performance in inhibitory control tasks. Zhao (2014) found that in the 6 weeks before the final exam of college students, their performance on Raven’s reasoning task (a task to measure fluid intelligence) declined. This decrease is equivalent to a 10 point decrease in IQ test scores. When students were asked, “How much free time do you have these days?” Their answers predicted their cognitive performance on Raven’s reasoning task. The more free time, the better cognitive performance. The researchers also induced participants’ perceived scarcity of time by initiating tasks, and the participants were divided into low time requirement conditions (non-time scarcity group) and high time requirement conditions (time scarcity group). Subsequently, the subjects were asked to complete a set of Raven’s reasoning tasks. The results showed that the scarcity group performed significantly lower on Raven’s reasoning tasks than the non-scarcity group. Zhao (2014) study also found that time scarcity can lead to a decrease in individuals’ memory and classification ability.

Moreover, the decrease in cognitive flexibility caused by perceived scarcity in current research may also be due to the concentration and transfer of attention. When individuals try to shift their attention from money-related issues to other tasks, they further deplete their executive functions (Baumeister et al., 1994; Mann and Ward, 2007), further exacerbating limited cognitive resources. This may also be one of the reasons for the results of the current research. In the current research, perceived scarcity also makes it difficult for individuals to transfer executive functions from scarcity-related issues to the current switching task, which in turn leads to poorer performance on the switching task and a temporary decrease in cognitive flexibility. The decline in cognitive flexibility further prevents individuals from switching quickly between tasks. Scarcity theory and empirical research on other executive functions also support this result (Mullainathan and Shafir, 2013). For example, Shah et al. (2018) published literature in Science showing that individuals experience more fatigue when faced with scarce opportunities for laboratory initiation, time, or economic scarcity. Subsequent attention tests found a worsened effect, with more borrowing without interest.

In the perspective of neural mechanisms, the ERP results to some extent support the impact of perceived scarcity on cognitive flexibility. Cued stimuli contained scarce or neutral words, which may have interfered with individuals’ EEG activity when processing cued stimuli. Therefore, we focused mainly on the ERP component of the undisturbed target-locked epochs. Previous research on cognitive control indicated that the amplitude of the P3 difference wave (repeat trials minus switch trials) also reflects switching costs. Compared to repeat trials, the P3 amplitude’s decrease in switch trials during the target-locked epochs is thought to reflect weak or unstable linkages between the stimulus and task setting in switch trials (Waszak et al., 2003; Tieges et al., 2007). For the P3 difference wave, which can reflect the switching cost, we found that the impact of perceived scarcity on cognitive flexibility was consistent in the EEG and behavioral results in the current study. Compared with the control group, perceived scarcity caused individuals to evoke smaller P3 amplitudes in the parietal cortex in switching trials and increased the amplitudes of P3 differential wave (repeat trials minus switch trials). This result reflects individual’s decreased cognitive flexibility in switching tasks, which in turn reduces their performance and increases the switching cost of reaction time in switching tasks. This is consistent with the results of existing studies that have found that the P3 component of the target-locked epochs is related to task rule updating and cognitive switching ability (West and Moore, 2005). Therefore, in the scarcity group the amplitude of the P3 differential wave’s increase in the parietal cortex reflects the detrimental impact of perceived scarcity on the degree of brain activation, resulting in a temporary decrease in cognitive flexibility. As a result, an individual’s performance in switching trials is worse, and the switching cost is higher.

## 5. Limitations

Laboratory experiments involving humans are often questioned due to a lack of external validity. Whether the experimental results can be extrapolated to other samples or populations, is one such question (Zhang and Yang, 2017). Combining laboratory experimental results with psychologically relevant theories can, however, partially address the issue of external validity (Duflo et al., 2007). Nevertheless, there is still a noteworthy difference between scarcity situational tasks, cognitive tasks created in a laboratory, and people’s daily life situations. Whether this can truly reflect the cognitive ability of individuals with high perceived scarcity in daily life, needs to be carefully considered. In the future, more field studies should be conducted to improve the external validity of the research results. Additionally, the current study was conducted on a college student population, which may have affected the generalizability of the results. Moreover, although the sample size met the statistical requirements, it was relatively small, and the validity of the conclusions needs to be verified. Therefore, repeating the results of our laboratory research in different groups would test the findings’ external validity and be a necessary link before the research results are promoted.

## 6. Conclusion

The current study found that the use of laboratory priming tasks can induce an individua’s perceived scarcity in an impactful manner, and the perceived scarcity of monetary resources depletes the individual’s cognitive resources, resulting in a temporary decline in participants’ cognitive flexibility. Specifically, in terms of neural activity, perceived scarcity led to an increase in the amplitude of the P3 differential wave (repeat trials minus switch trials) in the parietal cortex during the target-locked epochs in the switching task. In terms of behavioral outcomes, perceived scarcity led to poorer performance and a greater switching cost of reaction time in switching tasks.

## Data availability statement

The original contributions presented in the study are included in the article/supplementary material, further inquiries can be directed to the corresponding author.

## Ethics statement

The studies involving human participants were reviewed and approved by the Academic Ethics Committee of Wannan Medical College. The patients/participants provided their written informed consent to participate in this study.

## Author contributions

LH and XL: conceptualization, research methodology, and research design. FX and FL: conceptualization. All authors have read and approved the final version of the manuscript.

## Funding

This study was supported by the National Natural Science Foundation of China (Grants Nos. 71971103, 72164028), the Philosophy and Social Science Planning Project of Anhui Provincial (Grants Nos. AHSKF2021D21), the University Science Research Project of Anhui Provincial Department of Education (Grants Nos. 2022AH030119), and New era education quality engineering project (postgraduate education) of Anhui Provincial (2022lhpysfjd065).

## Conflict of interest

The authors declare that the research was conducted in the absence of any commercial or financial relationships that could be construed as a potential conflict of interest.

## Publisher’s note

All claims expressed in this article are solely those of the authors and do not necessarily represent those of their affiliated organizations, or those of the publisher, the editors and the reviewers. Any product that may be evaluated in this article, or claim that may be made by its manufacturer, is not guaranteed or endorsed by the publisher.
